# Supplementary material for: FSTruct: An F ST‐based tool for measuring ancestry variation in inference of population structure
Source: Mol Ecol Resour. 2022 Jul 20;22(7):2614–26. doi: 10.1111/1755-0998.13647 (PMC9544611; doi:10.1111/1755-0998.13647)
Supplement: Supplementary file 1 — Appendix S1 [file MEN-22-2614-s001.pdf]

Supplemental Information for:

FSTruct: an F_{ST}-based tool for measuring ancestry variation in inference of population structure

Maike L. Morrison, Nicolas Alcala & Noah A. Rosenberg

List of Figures

S1 Effect of α on sampling variability of Q matrix empirical means 2
S2 Effect of α on sampling variability of Q matrix empirical variances 3
S3 Effect of K on $F_{ST}/F_{ST}^{\max}$ 4
S4 Effect of the number of bootstrap samples on P-values for comparisons of bootstrap distributions of $F_{ST}/F_{ST}^{\max}$ between pairs of identical Q matrices 5
S5 Effect of the number of clusters (K) on P-values for comparisons of bootstrap distributions of $F_{ST}/F_{ST}^{\max}$ between pairs of identical Q matrices 6
S6 Effect of the number of individuals on P-values for comparisons of bootstrap distributions of $F_{ST}/F_{ST}^{\max}$ between pairs of identical Q matrices 7
S7 Effect of I on $F_{ST}/F_{ST}^{\max}$ 8

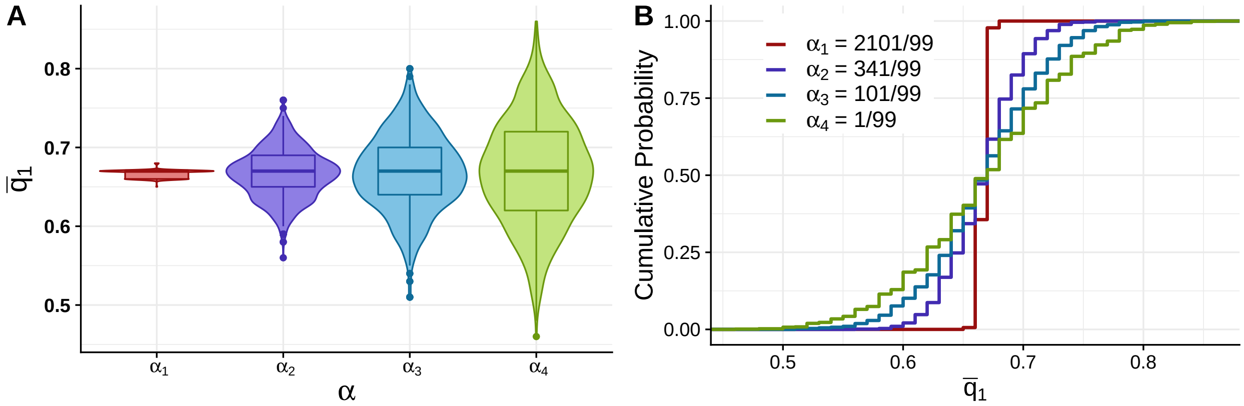

Figure S1: Effect of α on sampling variability of Q matrix empirical means. The plot answers the question, *"How different are the mean membership vectors of Q matrices simulated with identical Dirichlet parameter values?"*

We simulated Q matrices with the same parameter values used in Figure 4A: $I = 50$ individuals, Dirichlet mean $\boldsymbol{\lambda} = (\frac{2}{3}, \frac{1}{3})$, and α taking one of four values: $\alpha_1 = \frac{21901}{99}$, $\alpha_2 = \frac{341}{99}$, $\alpha_3 = \frac{101}{99}$, and $\alpha_4 = \frac{1}{99}$. We simulated 1,000 matrices for each value of α. For each simulated Q matrix, we computed the empirical mean of the largest ancestry coefficient, $\bar{q}_1 = \frac{1}{I}\sum_{i=1}^{I} q_1^{(i)}$, which we expect to be approximately equal to the parametric mean $\lambda_1 = \frac{2}{3}$ ($q_1^{(i)}$ is the proportion of individual i's ancestry assigned to cluster 1). For each value of α, we visualize the distribution of $\bar{q}_1$ across all 1,000 matrices simulated with that α.

(A) Boxplots and violin plots of the distributions of $\bar{q}_1$ across simulated matrices. **(B)** Empirical cumulative distribution functions of these distributions. Note that each point in these distributions is a single, independent, simulated Q matrix, not a bootstrap sample. We observe that the empirical means of the distributions are consistent across values of α. However, as α decreases (and the parametric variance $\sigma^2 = \lambda_1\lambda_2/(\alpha + 1)$ increases), the distributions get broader. This plot highlights the point that there is considerable sampling variability among Q matrices simulated with identical parameters, and that this sampling variability increases as the parametric variability increases. Thus it is unsurprising for Q matrices simulated with identical parameter values to often have significantly different bootstrap distributions of $F_{ST}/F_{ST}^{\max}$, as observed in Figure 4.

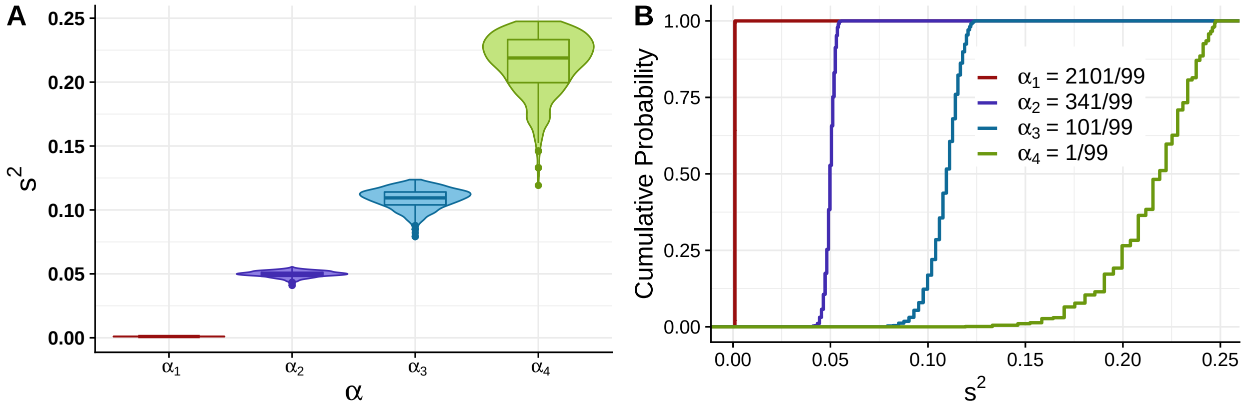

Figure S2: Effect of α on sampling variability of Q matrix empirical variances. The plot answers the question, *"For Q matrices simulated with identical Dirichlet parameter values, how different are the variances of sampled membership coefficients?"*

This plot contains the same simulation results as Figure S1, differing only in that it plots the distribution of the empirical variance $s^2 = \bar{q}_1\bar{q}_2/(\alpha+1)$ instead of the distribution of the empirical mean $\bar{q}_1 = \frac{1}{I}\sum_{i=1}^{I} q_1^{(i)}$ ($q_1^{(i)}$ is the proportion of individual i's ancestry assigned to cluster 1). We expect s^2 to be approximately equal to the parametric variance $\sigma^2 = \lambda_1\lambda_2/(\alpha+1)$; $\sigma^2 = 0.001$ for α_1, $\sigma^2 = 0.05$ for α_2, $\sigma^2 = 0.11$ for α_3, and $\sigma^2 = 0.22$ for α_4.

Like Figure S1, this plot highlights the point that, as the parametric variance used to simulate the Q matrices increases (as α decreases), the distributions of empirical variances of the simulated Q matrices are themselves more variable. Thus, it is unsurprising for Q matrices simulated with identical parameter values to often have significantly different bootstrap distributions of $F_{ST}/F_{ST}^{\max}$, as observed in Figure 4.

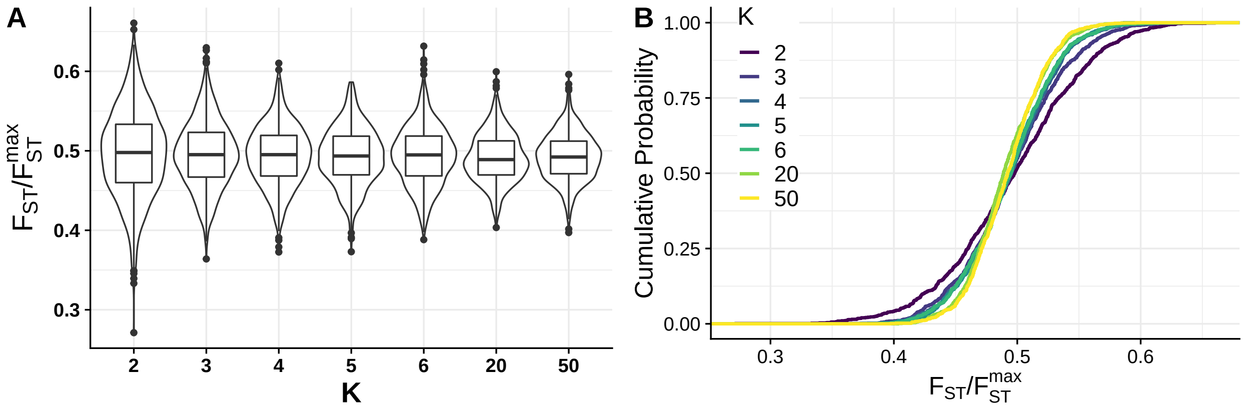

Figure S3: Effect of K on $F_{ST}/F_{ST}^{\mathbf{max}}$. This plot answers the question, *"Does the number of clusters K influence F_{ST}/F_{ST}^{max}?"*

For each of seven values of K, we simulated 1,000 Q matrices with $I = 50$ individuals, Dirichlet variability parameter $\alpha = 101/99$, and Dirichlet mean parameter $\boldsymbol{\lambda_K} = (\frac{2}{K+1}, \frac{1}{K+1}, \ldots, \frac{1}{K+1})$. The expected value of $F_{ST}/F_{ST}^{\max}$ for the $K = 2$ case, per eq. 2, is $1/(\alpha + 1) = 0.495$.

The simulated mean value of $F_{ST}/F_{ST}^{\max}$ was 0.497 for $K = 2$, 0.496 for $K = 3$, 0.494 for $K = 4$, 0.493 for $K = 5$ and $K = 6$, 0.491 for $K = 20$, and 0.492 for $K = 50$. Wilcoxon rank-sum tests for pairs of distributions produced $P = 1.000$ for most comparisons (exceptions were $P = 0.046$ between 2 and 20, $P = 0.129$ between 3 and 20, $P = 0.463$ between 4 and 20, and $P = 0.291$ between 2 and 50). These results suggest that the number of clusters, K, does not drive differences in $F_{ST}/F_{ST}^{\max}$ across Q matrices.

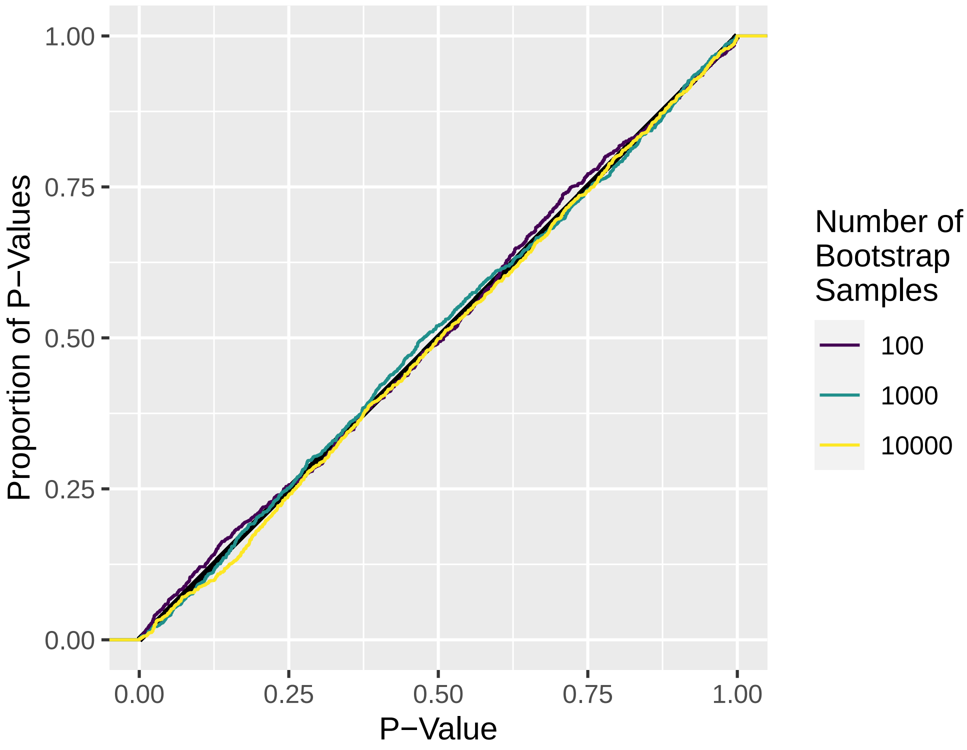

Figure S4: Effect of the number of bootstrap samples on P-values for comparisons of bootstrap distributions of $F_{ST}/F_{ST}^{\max}$ between pairs of identical Q matrices. The plot answers the question, *"Does the probability of rejecting the null hypothesis that two bootstrap distributions of F_{ST}/F_{ST}^{max} are identical depend on the number of bootstrap samples?"*

First, we chose a Q matrix. For each of three choices for the number of bootstrap samples (100, 1,000, 10,000), we generated 1,000 pairs of bootstrap distributions of $F_{ST}/F_{ST}^{\max}$ from that Q matrix—representing 1,000 instances in which no difference in variability exists between two matrices that are being compared. For each pair of distributions, we then computed the P-value for a Wilcoxon rank-sum test comparing the pair. Because the null hypothesis of no difference in variability between matrices holds in each of the 1,000 instances, the distribution of P-values is expected to be uniform, irrespective of the number of bootstrap samples.

Indeed, for all three choices for the number of bootstrap samples, the empirical cumulative distribution function (ECDF) of 1,000 P-values was comparable to the uniform distribution, depicted as a black diagonal line (Kolmogorov-Smirnov test, $p = 0.35$ for 100 samples, $p = 0.49$ for 1,000 samples, $p = 0.31$ for 10,000 samples). We conclude from the figure that the ECDF of P-values from Wilcoxon rank-sum tests comparing pairs of bootstrap distributions of $F_{ST}/F_{ST}^{\max}$ for identical Q matrices is uniform. The same underlying Q matrix was considered for all pairs of bootstrap distributions: a matrix with $I = 50$ individuals (rows) generated with a Dirichlet distribution with mean $\boldsymbol{\lambda} = (\frac{2}{3}, \frac{1}{3})$ and $\alpha = 101/99$ (the 23rd α value explored in Figure 3 and the third value depicted in Figure 4).

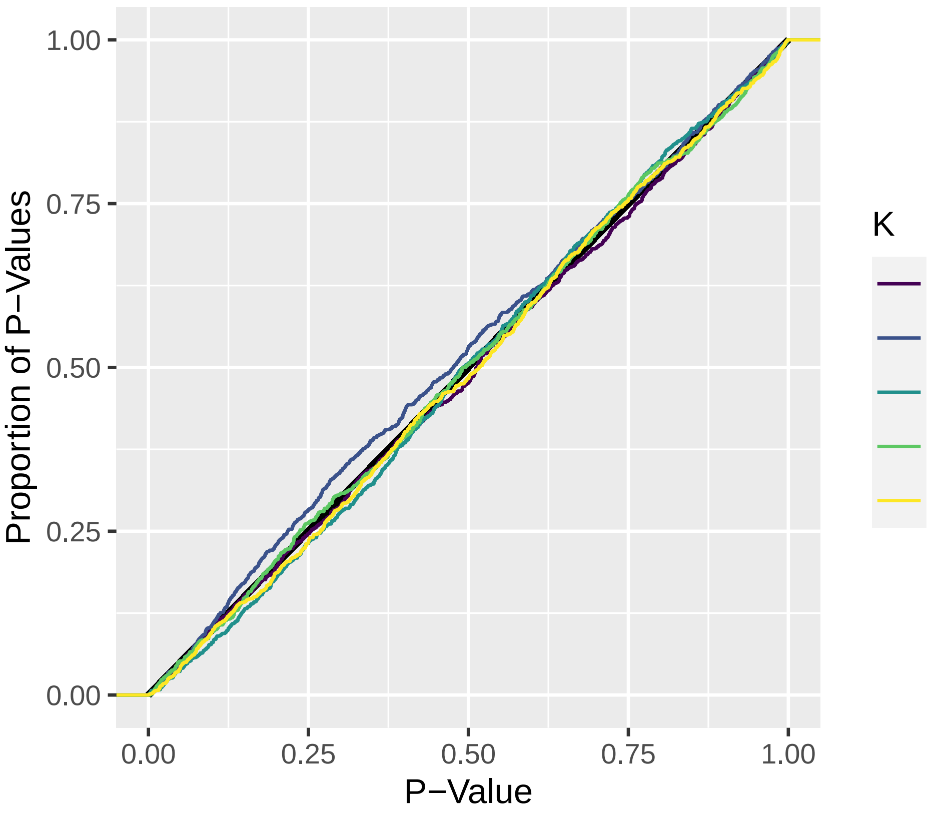

Figure S5: Effect of the number of clusters (K) on P-values for comparisons of bootstrap distributions of $F_{ST}/F_{ST}^{\max}$ between pairs of identical Q matrices. The plot answers the question, *"Does the probability of rejecting the null hypothesis that two bootstrap distributions of F_{ST}/F_{ST}^{max} are identical depend on the number of clusters (columns) in the Q matrices?"*

First, we simulated five Q matrices, each with a different number of clusters: $K = 2, 3, 4, 5$ or 6. We set $\boldsymbol{\lambda}_K = (\frac{2}{K+1}, \frac{1}{K+1}, \ldots, \frac{1}{K+1})$ (the same means used in Figure S3). For $K = 2$, this $\boldsymbol{\lambda}_K$ is the same mean used elsewhere in our analyses ($\boldsymbol{\lambda} = (\frac{2}{3}, \frac{1}{3})$). The other Dirichlet parameters were held constant ($I = 50$ individuals, $\alpha = \frac{101}{99}$). For each of the five Q matrices, we generated 1,000 pairs of bootstrap distributions of $F_{ST}/F_{ST}^{\max}$, each with 1,000 sampled matrices per distribution. For each pair of distributions, we then computed the P-value for a Wilcoxon rank-sum test comparing the pair. Because the null hypothesis of no difference in variability between matrices holds in each of the 1,000 instances, the distribution of P-values is expected to be uniform.

Indeed, for all five choices for the number of clusters, the empirical cumulative distribution function (ECDF) of P-values was comparable to the uniform distribution, depicted as a black diagonal line (Kolmogorov-Smirnov test, $P = 0.567$ for $K = 2$, $P = 0.041$ for $K = 3$, $P = 0.320$ for $K = 4$, $P = 0.868$ for $K = 5$, $P = 0.716$ for $K = 6$). We conclude from the figure that the ECDF of P-values from Wilcoxon rank-sum tests comparing pairs of bootstrap distributions of $F_{ST}/F_{ST}^{\max}$ for identical Q matrices is uniform and does not depend on the number of clusters in the analysis.

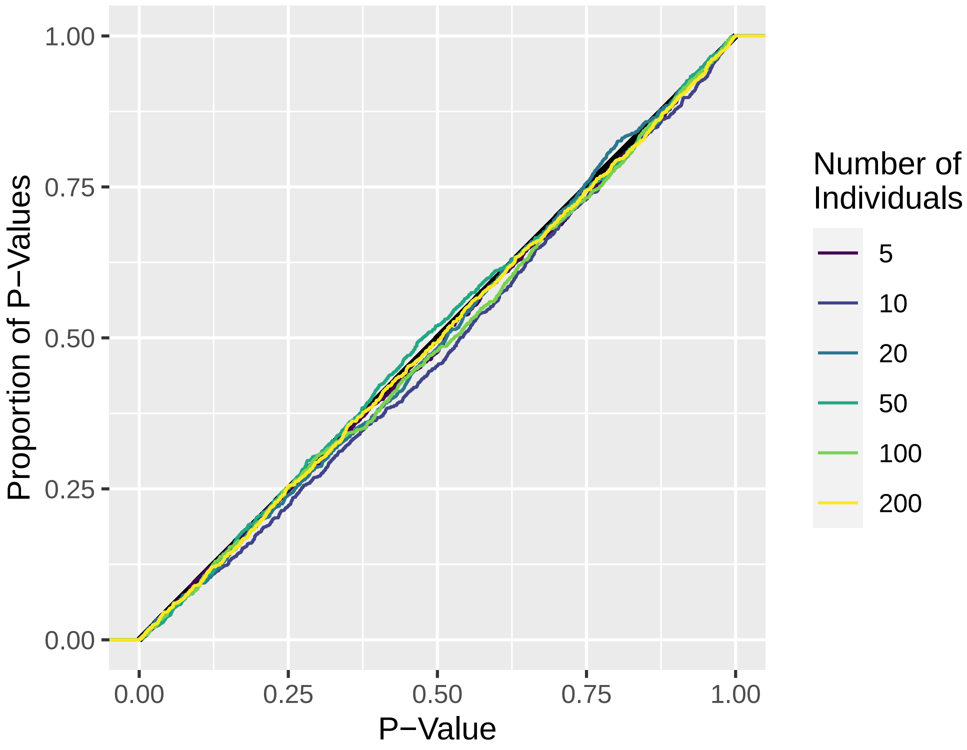

Figure S6: Effect of the number of individuals on P-values for comparisons of bootstrap distributions of $F_{ST}/F_{ST}^{\max}$ between pairs of identical Q matrices. The plot answers the question, *"Does the probability of rejecting the null hypothesis that two bootstrap distributions of F_{ST}/F_{ST}^{max} are identical depend on the number of individuals (rows) in the Q matrices?"*

First, we simulated six Q matrices, each with a different number of individuals: $I = 5, 10, 20, 50, 100,$ or 200. The other Dirichlet parameters were held constant ($\boldsymbol{\lambda} = (\frac{2}{3}, \frac{1}{3})$, $\alpha = \frac{101}{99}$). For each of the six Q matrices, we generated 1,000 pairs of bootstrap distributions of $F_{ST}/F_{ST}^{\max}$, each with 1,000 sampled matrices per distribution. For each pair of distributions, we then computed the P-value for a Wilcoxon rank-sum test comparing the pair. Because the null hypothesis of no difference in variability between matrices holds in each of the 1,000 instances, the distribution of P-values is expected to be uniform.

Indeed, for all six choices for the number of individuals, the empirical cumulative distribution function (ECDF) of P-values was comparable to the uniform distribution, depicted as a black diagonal line (Kolmogorov-Smirnov test, $P = 0.567$ for $I = 5$, $P = 0.012$ for $I = 10$, $P = 0.383$ for $I = 20$, $P = 0.490$ for $I = 50$, $P = 0.177$ for $I = 100$, $P = 0.904$ for $I = 200$). We conclude from the figure that the ECDF of P-values from Wilcoxon rank-sum tests comparing pairs of bootstrap distributions of $F_{ST}/F_{ST}^{\max}$ for identical Q matrices is uniform and does not depend on the number of individuals analyzed.

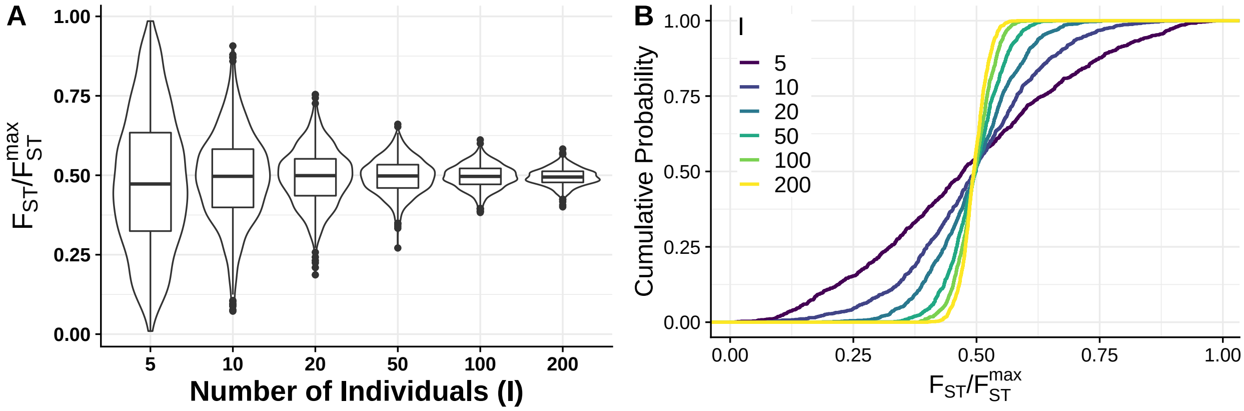

Figure S7: Effect of I on $F_{ST}/F_{ST}^{\mathbf{max}}$. This plot answers the question, *"Does the number of individuals or rows of the Q matrix, I, influence F_{ST}/F_{ST}^{max}?"*

For each of six values of I (5, 10, 20, 50, 100, and 200), we simulated 1,000 Q matrices with Dirichlet variability parameter $\alpha = 101/99$, and Dirichlet mean parameter $\boldsymbol{\lambda} = (\frac{2}{3}, \frac{1}{3})$ (the same parameters used in Figure S6). The expected value of $F_{ST}/F_{ST}^{\max}$ for the $K = 2$ case, per eq. 2, is $1/(\alpha + 1) = 0.495$.

The mean value of $F_{ST}/F_{ST}^{\max}$ was 0.482 for $I = 5$, 0.493 for $I = 10$, 0.495 for $I = 20$, 0.497 for $I = 50$, 0.496 for $I = 100$, and 0.495 for $I = 200$. Wilcoxon rank-sum tests for pairs of distributions produced $P = 1.000$ for most comparisons (exceptions were $P = 0.666$ between 5 and 10, $P = 0.194$ between 5 and 20, $P = 0.065$ between 5 and 50, $P = 0.065$ between 5 and 100, and $P = 0.065$ between 5 and 200). Although the means of the distributions do not depend on the number of individuals, the standard deviations of the distributions decrease with I ($s = 0.214$ for $I = 5$, $s = 0.140$ for $I = 10$, $s = 0.089$ for $I = 20$, $s = 0.055$ for $I = 50$, $s = 0.038$ for $I = 100$, $s = 0.027$ for $I = 200$). These results suggest that although the number of individuals, I, does not drive differences in the mean value of $F_{ST}/F_{ST}^{\max}$ for fixed α, it does influence the variability.
